# Evidence for strong, widespread chlorine radical chemistry associated with pollution outflow from continental Asia

**DOI:** 10.1038/srep36821

**Published:** 2016-11-15

**Authors:** Angela K. Baker, Carina Sauvage, Ute R. Thorenz, Peter van Velthoven, David E. Oram, Andreas Zahn, Carl A. M. Brenninkmeijer, Jonathan Williams

**Affiliations:** 1 Max Planck Institute for Chemistry, Mainz, Germany; 2 Royal Netherlands Meteorological Institute (KNMI), De Bilt, the Netherlands; 3 National Centre for Atmospheric Science, University of East Anglia, Norwich, United Kingdom; 4 Karlsruhe Institute of Technology, Karlsruhe, Germany

## Abstract

The chlorine radical is a potent atmospheric oxidant, capable of perturbing tropospheric oxidative cycles normally controlled by the hydroxyl radical. Significantly faster reaction rates allow chlorine radicals to expedite oxidation of hydrocarbons, including methane, and in polluted environments, to enhance ozone production. Here we present evidence, from the CARIBIC airborne dataset, for extensive chlorine radical chemistry associated with Asian pollution outflow, from airborne observations made over the Malaysian Peninsula in winter. This region is known for persistent convection that regularly delivers surface air to higher altitudes and serves as a major transport pathway into the stratosphere. Oxidant ratios inferred from hydrocarbon relationships show that chlorine radicals were regionally more important than hydroxyl radicals for alkane oxidation and were also important for methane and alkene oxidation (>10%). Our observations reveal pollution-related chlorine chemistry that is both widespread and recurrent, and has implications for tropospheric oxidizing capacity, stratospheric composition and ozone chemistry.

During boreal winter, deep convection over the Western Tropical Pacific is the dominant pathway for the transport of surface air to the tropical tropopause layer (TTL, 13–17 km) and into the stratosphere^1^,^2^,^3^. As such, tropospheric composition and chemistry there plays a disproportionately large role in dictating stratospheric composition. Although research activity in the region has increased in recent years airborne observations remain scarce, particularly in the upper part of the free troposphere around the TTL. The CARIBIC (Civil Aircraft for the Regular Investigation of the atmosphere Based on an Instrument Container) dataset is unique in that it covers multiple years and seasons in this particular location and altitude range. Recent campaigns and modeling efforts have concentrated mainly on characterizing regional sources of short-lived halocarbons and quantifying their stratospheric loadings^4^, with most of the focus being on naturally occurring brominated compounds and, more recently, anthropogenic chlorocarbon emissions^5^. There is also increased interest in the role of Br and I as oxidants, however, the potential of Cl as an oxidant in the region has so far not been addressed.

Even when present at low levels, Cl radicals can have a profound impact on tropospheric oxidation and radical cycling. Cl chemistry can significantly impact levels of tropospheric ozone, a greenhouse gas that is also a precursor for the OH radical, destroying it in clean environments and enhancing its formation under polluted conditions. Additionally, much faster rates of reaction, in comparison to OH, allow Cl radicals to expedite oxidation of climate-active gases, such as methane (CH_4_) and dimethyl sulphide. The introduction of reactive chlorine into the troposphere is understood to occur mainly via heterogeneous processes on particulate surfaces, typically sea salt, which release particle bound chlorine into the gas phase^6^,^7^. The main mechanisms for chlorine release are (i) displacement of particulate hydrochloric acid by nitric or sulfuric acid, (ii) photochemical production of molecular chlorine on aerosol surfaces, and, in polluted areas with high levels of nitrogen oxides (NO_x_), (iii) formation of nitryl chloride (ClNO_2_) by uptake of N_2_O_5_ on the surface of chlorine containing aerosols at night^8^. Once thought to be restricted to the marine and polar boundary layer, recent observations have found that chlorine radicals also play a significant role in coastal urban and even mid-continental areas^9^,^10^, believed to be a result of mechanism (iii). While Cl radical concentrations approaching 10^6^ Cl cm^−3^ have been reported^9^, the global average marine boundary layer concentration is estimated to be about 10^3^ Cl cm^−3^, with Cl chemistry generally considered minor on the global scale.

Over two consecutive winter seasons (November 2012 to March 2013 and November 2013 to January 2014) the IAGOS-CARIBIC observatory^11^ was deployed monthly on round-trip commercial flights between Bangkok, Thailand and Kuala Lumpur, Malaysia (Table 1). Observations were made at cruise altitudes between 9 and 12 km and included the collection of air samples, which were analyzed in the laboratory for over 60 trace gases, including a suite of 20 non-methane hydrocarbons (NMHCs)^12^. Supporting meteorological analyses^13^,^14^ indicate that the air masses encountered had passed over the South China Sea and/or tropical Western Pacific (Fig. 1), except during March 2013, when air masses were from the Indian Ocean. Here we investigate the surprising signals observed in the hydrocarbons during six separate flights with air masses traceable to the South China Sea (November 2012, December 2012, February 2013, November 2013, December 2013 and January 2014; Table 1). These signals were distinctly different from those previously observed at similar altitudes and latitudes elsewhere in the global dataset and indicative of chlorine radical chemistry.

## Results

During November and December of both 2012 and 2013 unusually high ratios of i-butane to n-butane were observed, which were not seen on the same route in the following January or February (Fig. 2A). The atmospheric ratio of these compounds typically falls within a range of 0.3 to 0.6 i-butane/n-butane^15^,^16^, and observations have largely fallen within this range in a variety of environments, from polluted urban areas to clean background sites. While some point sources are known to have emission ratios falling outside of this range, they are relatively scarce, and the isomeric ratio is largely determined by the ratio in the largest anthropogenic sources (e.g. vehicular emissions, fossil fuels) and in biomass burning emissions. Although observations of NMHCs are few in this region, there is no evidence in the literature that butane emission ratios deviate substantially from ratios observed elsewhere, and furthermore no evidence to support widely varying seasonal emissions. Therefore, it is unlikely that unusual source ratios are at play on such a wide scale.

Near-equal loss rates via reaction with the hydroxyl radical (OH) causes i-butane/n-butane to vary little with transport. However, because the rate of removal of n-butane by the chlorine radical (Cl) is ~50% faster than that of i-butane, Cl oxidation causes the i-butane/n-butane ratio to increase. Ratios in November and December were up to 43% higher than in January and February, and higher still than observed along other CARIBIC routes (Fig. 2A, grey crosses). The higher variability of the highlighted data compared to that taken elsewhere (see grey points in Fig. 2A) is likely due to the colocation of strong convection and higher levels of pollution at the surface. These observed large deviations are well beyond the measurement uncertainty (see Method) and imply significant hydrocarbon losses due to oxidation by Cl in this region.

Direct measurements of chlorine radicals are not currently possible, making indirect methods necessary to estimate Cl abundance and to gauge its importance relative to OH in tropospheric photochemistry. To access this information we have examined variations in the relationships of three NMHCs having suitable rates of reaction with Cl and OH^17^. Losses of an atmospheric species, A, attributable to a single oxidant, X, can be described by the equation


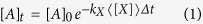


where [A]_t_ is the concentration at time t, [A]_0_ is the initial concentrations, k_X_ is the rate of reaction with oxidant X and [X] is the mean concentration of the oxidant during the transport time, Δt. Equation 1 can be rearranged to solve for any of the above parameters, and is often used as the basis for so-called “NMHC photochemical clock” methods^17^,^18^,^19^,^20^, which are frequently employed to investigate transport times (Δt) or oxidant concentrations. Given the large variability in NMHC concentrations and corresponding uncertainty in their initial levels, these methods more commonly rely on NMHC ratios, comparing observed ratios with typical emission ratios, which tend to lie within narrow ranges and are relatively well-known. In order to reduce the influence of mixing, observed ratios, also referred to as enhancement ratios, are determined from the slopes of the linear least squares fit to the data (Fig. 2, Table 1). Changes in NMHC ratios with processing can be described by combining Equation 1 for two compounds, A and B (expressed here in terms of the natural logarithm):


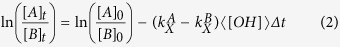


The further combination of Equation 2 for two different oxidants and NMHC pairs results in a time-independent expression used to derive the relative contributions of OH and Cl:


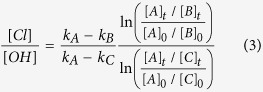


In Equation 1, A and B represent a NMHC pair having approximately equal rates of reaction with OH but different rates of reaction with Cl (Cl reactive pair), while A and C have approximately equal reaction rates with Cl but different reaction rates with OH (OH reactive pair). By comparing observed ratios (*t*) to initial ratios (*0*), it is possible to quantify the mean ratio of Cl to OH encountered by the air mass between emission at the Earth’s surface and sampling at 10–12 km. It should be noted that the ratio derived is an average integrated over the transport path and an influence of in-mixing of other aimasses cannot be ruled out. In this case, in-mixing is most likely to be from the relatively clean and photochemically aged Pacific free troposphere and boundary layer. Since such air will have been predominately oxidized by OH radicals, in-mixing will have the effect of biasing the data towards OH making estimates of the chlorine abundance conservative.

Here we use i-butane and n-butane as the Cl reactive pair, and propane and i-butane as the OH reactive pair (Table 2), and comparison of these ratios already shows qualitatively the influence of Cl (Fig. 2B). If OH alone acts on an air parcel, i-butane/propane would decrease with processing, while i-butane/n-butane remains constant; conversely, Cl chemistry alone would cause i-butane/n-butane to increase with i-butane/propane staying the same. As OH is omnipresent in the sunlit troposphere, changes in both ratios are expected when Cl is also present, and this behavior is indeed observed in November and December of both years. In contrast, the January and February variability in i-butane/n-butane is small (vertical axis in Fig. 2B), indicating that Cl chemistry was negligible at this time, see discussion. Analogous assessments with combinations of higher hydrocarbons were not possible as these more reactive species were mostly below the 1 pptv detection limit in this altitude range.

Using Equation 3 we have estimated the radical ratio, [Cl]:[OH], during each month in order to constrain the relative importance of Cl as an oxidant (Fig. 3A). The role of Cl radicals generally becomes significant at [Cl]:[OH] of about 10^−3^, when Cl radical concentrations (usually lower than OH) and rate coefficients (usually faster than with OH) combine so that Cl-initiated oxidation competes with that of OH^7^,^21^. We therefore express the ratio as Cl:10^3^ OH, such that values >1 indicate relative levels of Cl significant in oxidative processes. In the absence of corresponding surface measurements, we have applied literature values of 0.6 and 0.3 for i-butane/n-butane and i-butane/propane, respectively, derived from observations made in the Pearl River Delta region of China^22^, and note that these values are nearly identical to observations made in other urban areas throughout Asia^23^,^24^. Our observations in November and December of both years correspond to [Cl]:[OH] between 9.1 ± 2.3 and 16.0 ± 2.3 Cl:10^3^ OH (errors denote 95% confidence interval based on the error of the slopes of the correlations) (Fig. 3A). Therefore assuming a value for OH of 1 × 10^6^ molecules cm^−3^, then Cl radicals were present at levels between 0.9–1.6 × 10^4^ molecules cm^−3^. It should be noted, however, that this is the transit average and if the chlorine signature is created when the emissions are fresh (see discussion) then very much higher chlorine radical concentrations (approaching 10^6^ molecules cm^−3^) are present. The largest uncertainty in these estimates lies in the choice of initial ratios, however, applying values that span the range of typical reported ratios for these species we find that even the most conservative estimates correspond to [Cl]:[OH] >1 Cl:10^3^ OH. It should be noted that this method provides an estimate of [Cl]:[OH] integrated over the history of the air mass and does not fully account for the mixing in of air masses having different photochemical histories, although this influence is reduced through the use of enhancement ratios. As mixing most likely results in the introduction of air masses having been acted on exclusively by OH, the result would be an underestimation of [Cl]:[OH] in the initial air mass. Thus our measurements indicate the presence of significant quantities of Cl radicals in November and December.

To explore the significance of Cl as a tropospheric oxidant we have used the results derived above to calculate the rate of removal by Cl relative to removal by OH during each month for four classes of hydrocarbons important in this region: methane, alkanes, alkenes, and short-lived halocarbons (Fig. 3B, Table 2). The rate of reaction between an NMHC and either OH or Cl is determined for 298 K and 1 atm as it is assumed that the chlorine chemistry occurs near the ground (see discussion). Oxidant-specific removal rates are determined as the product of the rate coefficient, k_X_, and oxidant concentration, [X]; therefore, the relative rate of removal by Cl to removal by OH can be determined as


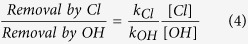


Relative removal rates were calculated for methane and, in the case of alkanes, alkenes and short-lived halocarbons, as the average value for a set of representative species (Table 2).

In November and December, when we found [Cl]:[OH] is an order of magnitude greater than in January and February, Cl is the dominant sink for alkanes, with removal rates up to twice as fast as by reaction with OH (Fig. 3B). Removal of methane and alkenes by Cl is also large, accounting for up to 20% and 13% of methane and alkene removal, respectively. Conversely, Cl is only a minor sink (<5%) for directly emitted short-lived halocarbons, the abundances and fates of which have been given much recent attention in this region owing to their uncertain role in stratospheric ozone loss^25^. Therefore we find that regional Cl chemistry has a strong potential for formation of long-lived chlorinated products via alkene oxidation, while doing little to decrease the abundances of primary halocarbons.

## Discussion

**Figure 1:**
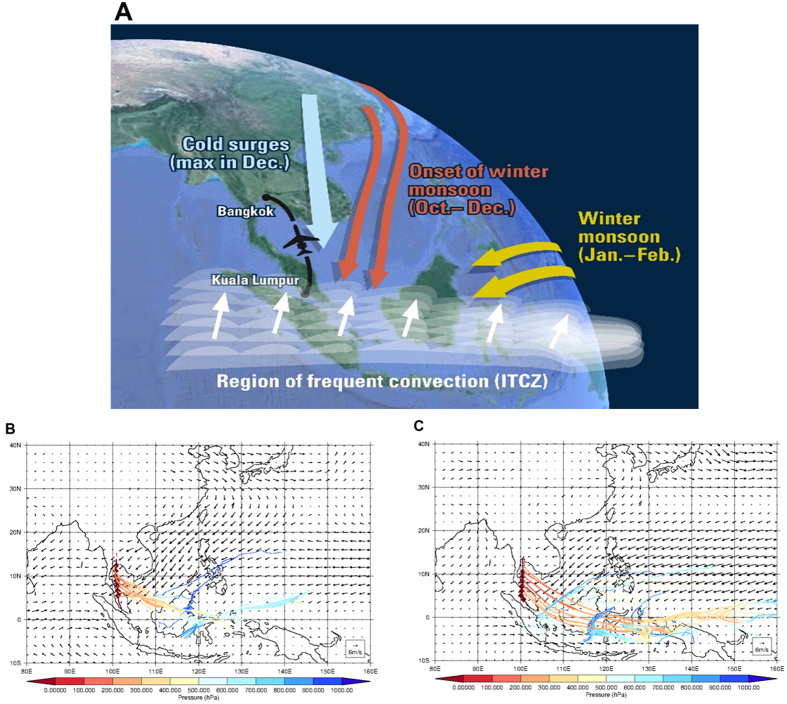
(**A**) Schematic of prevailing meteorological conditions over Southeast Asia and the Maritime Continent during the Northeast (winter) Monsoon (background map from Google Earth Map data: Google, DigitalGlobe). (**B**,**C**) 8-day backwards air mass trajectories for air samples collected on flights to Kuala Lumpur during December 2013 (**B**) and February 2013 (**C**), overlaid on mean surface (950 hPa) winds for the preceding 10 days. Data visualizations produced using IDL8.5 Excelis Visual Information Solutions, Boulder, Colorado, USA. http://www.harrisgeospatial.com/ProductsandSolutions/GeospatialProducts/IDL.aspx.

**Figure 2:**
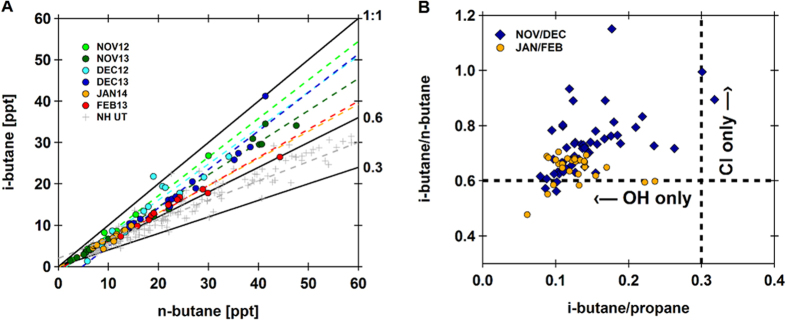
(**A**) Correlation plots of i-butane vs. n-butane observed at 9–12 km during flights between Bangkok and Kuala Lumpur (monthly data) and for all CARIBIC flights in the Northern Hemisphere Upper Troposphere (NH UT) between November and March (grey crosses). Dashed lines represent the linear least squares fit to the data and solid lines represent typical minimum and maximum emission ratios (0.3 and 0.6) and the 1:1 line. Fit data can be found in the Table 1. (**B**) Relationship between the i-butane to n-butane ratio and i-butane to propane ratio during November and December of 2012 and 2013 (blue diamonds), and January 2014 and February 2013 (orange circles).

**Figure 3:**
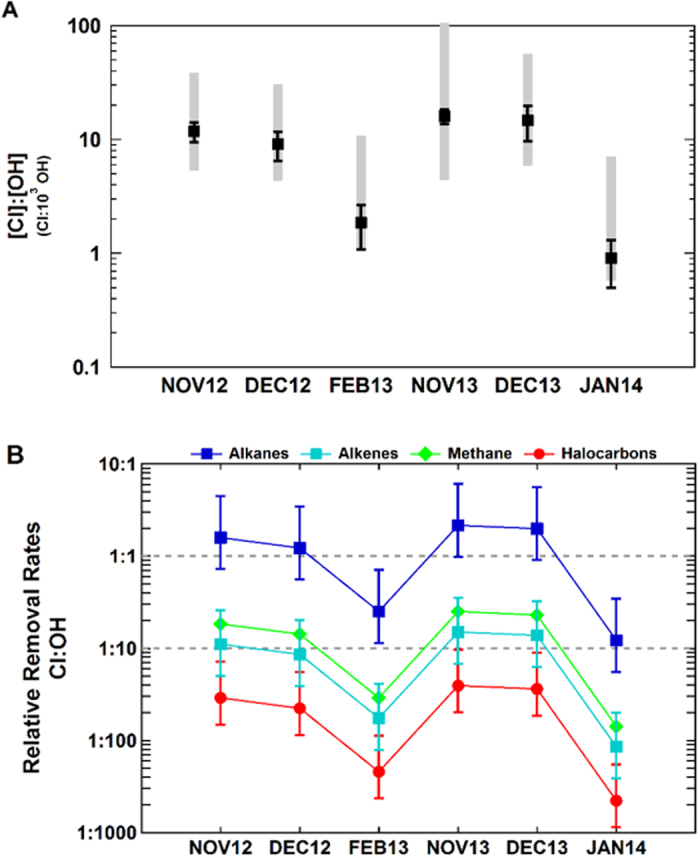
(**A)** Mean (black squares) and range (grey bars) of [Cl]:[OH] calculated for each month (see text). Error bars denote 95% confidence interval for the mean. (**B)** Mean relative removal rates by Cl and OH for methane, alkanes, alkenes and short-lived halocarbons (see Table 2), estimated for each month, with error bars indicating the spread. The dashed lines represent where removal by Cl and OH are equal (upper line) and where removal by Cl is 10% that of OH (lower line).

**Figure 4:**
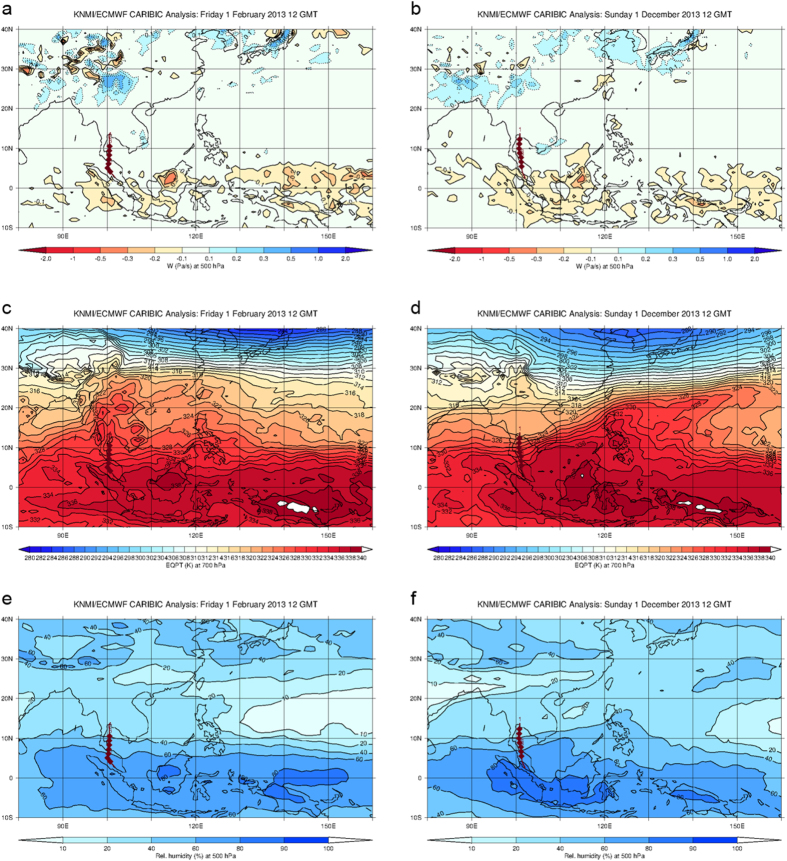
Monthly mean plots of vertical winds speeds at 500 hPa (**a**,**b**), equivalent potential temperature at 700 hPa (**c**,**d**), and relative humidity at 500 hPa (**e**,**f**), from the ECMWF archive. Figures on the left (**a,c,e**) are for February 2013 and figures on the right (**b,d,f**) are for December 2013. These parameters are used to identify the most active convective spots, and therefore the most likely regions of uplift, for the region during these months. More negative vertical wind speeds (upward movement) and higher potential temperatures (warmer/more moist air) are indicative of a greater potential for convective activity, while relative humidity at 500 hPa is an indication of which air masses have most recently been influenced by (more humid) air, presumably from the boundary layer. Data visualizations produced using IDL8.5 Excelis Visual Information Solutions, Boulder, Colorado, USA. http://www.harrisgeospatial.com/ProductsandSolutions/GeospatialProducts/IDL.aspx.

Knowledge of the transport pathways of the sampled air masses is critical to identifying the source of these Cl radicals. During boreal winter, regional circulation patterns are controlled by the Northeast Monsoon (November – March), which, at the surface, transports air masses over the South China Sea to equatorial Southeast Asia. Here they can be lofted to higher altitudes via the frequent strong convection present there^26^ with overall transport times between 3 and 7 days. Transport from East Asia is strongest at the onset of the monsoon and weakens in later months, as surface winds change to bring more air from the Western Pacific (Fig. 1A). Prevailing wind patterns are punctuated by incidences of rapid meridional transport, or cold surges, which expedite the movement of pollution to the south^27^. As the height of peak convective outflow (~12–13 km) is close to our CARIBIC sampler flight altitudes the probability of encountering convective outflow is very high^2^. Measurements were taken at 10–12 km, well below the local tropopause (16–18 km) and there was no indication in the ozone or RH data of stratospheric influence. Thus, our data will to a large extent reflect the composition and chemistry of convected air from the surface. These transport patterns are evident for the observation period (Fig. 1B,C), and vertical wind speeds combined with relative humidity and equivalent potential temperature support the presence of convection, with maximum intensity over Borneo (Fig. 4). Pollution export was evident in our observations during November and December, with typical urban/industrial tracers, such as dichloromethane, being well above background^28^. This strongly suggests that the effect is associated with pollution outflow.

Given the association with polluted air masses, ClNO_2_ seems a likely agent for producing the observed chlorine radical signatures. Previous observations have shown ClNO_2_ formation to be an important process in coastal urban areas, where anthropogenic emissions interact with sea salt aerosols^21^,^29^. Moreover, there is growing observational support for mid-continental ClNO_2_ sources^30^, and modelling studies predict strong ClNO_2_ formation over Southeast Asia during winter^31^. Recently, extremely high levels of Cl-NO_2_ have been reported in the plumes of Chinese megacities^32^,^33^,^34^. Estimated levels in the residual layer based on their measurement data ranged from 1.7 to over 4 ppb leading to chlorine radical production rates in excess of 1 ppb hr^−1^. In the light of this finding it seems likely that mechanism iii is the most likely source of the chlorine radical chemistry in our study. It should be noted that while Cl chemistry is most significant in November and December, the source regions of the air masses were different in January – March. High Cl-NO_2_ concentrations have been measured at the ground in the Pearl river delta region in both winter^34^ and summer^32^,^33^ suggesting that the apparent seasonality in the signal observed at altitude stems from the meteorology rather than a variation of source strength.

In comparison to previous measurements made in the United States (Texas^35^ and California^21^) the recently reported levels of Cl-NO_2_ and NOx are significantly higher^32^. Rather than providing only a transient surge of Cl radicals at sunrise, levels of Cl-NO_2_ were sustained for much longer and even grew for four hours after dawn. The high levels of NO_2_ present can serve to suppress OH (through HNO_3_ formation) and in doing so prevent the masking of Cl radical chemistry by secondary OH production shown by Young *et al*.^21^. Therefore the combination of high sustained ClNO_2_ and NO_2_ levels over the mainland China source region may explain why the chlorine chemistry is so profound that it can be observed by our aircraft at 10 km altitude.

We note that a ClNO_2_ source of Cl radicals would also serve as a source of nitrate (NO_3_) radicals, which also react with the NMHCs discussed here. NO_3_ would also be expected to play a role in nighttime oxidation in regions having high levels of NO_x_ and O_3_ (regardless of the presence of ClNO_2_), as would be expected for polluted air masses originating in continental Asia. However, reactions of alkanes with NO_3_ proceed over one thousand times more slowly than with Cl (or OH) and are on the order of 10^−17^–10^−16^ cm^3^ molec^−1^ s^−1^ and would not be expected to be a dominant loss mechanism. More significantly, the reaction of NO_3_ with i-butane is faster than with n-butane, so reaction with NO_3_ would cause the ratio of i-butane/n-butane to decrease over time. This again suggests that the significant Cl radical abundances we have derived from our data are likely underestimates.

In addition to the influence on tropospheric oxidative cycles, Cl radical chemistry occurring in these polluted air masses would be expected to result in the formation of secondary chlorinated species via reaction with the alkenes that are abundant in anthropogenic pollution. While oxidation of alkanes by Cl occurs via the same (hydrogen abstraction) process as by OH, resulting in more rapid formation of oxygenated products (e.g. formaldehyde, acetone)^36^, oxidation of alkenes proceeds via addition of Cl and results in the formation of chlorinated products (e.g. formyl chloride and chloroacetone)^37^,^38^, creating a relatively long-lived (>1 month) Cl reservoir. These chlorinated secondary products of hydrocarbon oxidation by OH are not included in current inventories, which so far only consider the products of halocarbon oxidation by OH^5^. Nonetheless, these *in-situ* chlorinated products can enter the stratosphere and contribute to the total stratospheric reactive chlorine budget, which is key to stratospheric ozone depletion but not yet closed^39^.

In the Western Tropical Pacific region, where transport patterns facilitate global redistribution of pollutants and persistent deep convection creates a fast-track to the stratosphere, the regional chlorine chemistry described here can lead to global scale impacts. Chlorine chemistry on the scale suggested by our observations would perturb significantly both the composition and radical abundances of the free troposphere, thereby affecting concentrations of greenhouse gases such as methane. Additionally, oxygenated and chlorinated species produced via Cl chemistry, can be transported to and across the TTL. These findings also point to a potential mechanism for chlorine, possibly extracted from sea salt by anthropogenic NO_x_ pollution via ClNO_2_ formation, to enhance atmospheric oxidation capacity and enter the stratosphere as chlorinated products of hydrocarbon oxidation, exacerbating ozone depletion.

## Methods

### Air sample collection and NMHC analysis

The CARIBIC scientific payload consists of 15 measurement systems, is fully automated, and carries out *in-situ* trace gas and aerosol measurements, as well as remote sensing by DOAS and the collection of aerosol and whole air samples^11^. Supporting meteorological analyses are based on the TRAJKS model^13^,^14^ and included 8-day backward trajectories for the air samples. During flights between Bangkok and Kuala Lumpur air samples were collected in glass sampling flasks, with 7 samples collected on the way to Kuala Lumpur and 7 on the return to Bangkok. The flasks are 2.7 L in volume and are filled to ~4.5 bar at pre-determined, 8 minute (~125 km) intervals with collection times of about 30 s (~7 km). Upon return of the container to the institute in Mainz the air samples are removed and analyzed in the laboratory for NMHCs. This analysis uses an HP-6890 gas chromatograph (GC) coupled with a flame ionization detector (FID), where, prior to analysis, a 1l (STP) aliquot of sample is pretreated by drying followed by cryogenic pre-concentration and cryo-focusing^12^. A suite of 20 compounds is measured, consisting of the C_2_-C_8_ alkanes, ethyne and the BTEX aromatics (benzene, toluene, ethylbenzene and the xylenes). Calibration of NMHCs is based on individual compound response factors determined from analysis of a synthetic mixture of NMHCs (accuracy of ±2%) purchased from the National Physical Laboratory (NPL, United Kingdom). The compounds discussed herein (n-butane, i-butane and propane) have analytical precisions and accuracies of less than 1%, limits of detection of 1 ppt, and overall uncertainties of less than 5%. As of this writing over 6000 CARIBIC whole air samples have been measured on this system.

## Additional Information

**How to cite this article**: Baker, A. K. *et al*. Evidence for strong, widespread chlorine radical chemistry associated with pollution outflow from continental Asia. *Sci. Rep.* **6**, 36821; doi: 10.1038/srep36821 (2016).

**Publisher’s note**: Springer Nature remains neutral with regard to jurisdictional claims in published maps and institutional affiliations.

## Supplementary Material

Supplementary Information

## Figures and Tables

**Table 1:** Flight dates and fit information for i-butane/n-butane correlations shown in Fig. 2.

**Flight Date**	**i-butane/n-butane**	
	**slope**	**R**^**2**^
22 November 2012	0.93	0.99
13 December 2012	0.87	0.89
21 February 2013	0.587	0.97
09 November 2013	0.78	0.99
05 December 2013	0.93	0.92
18 January 2014	0.65	0.88

**Table 2:** Rates of reaction with OH and Cl and classification for Fig. 3 for various hydrocarbons^32^,^36^ at 298 K and 1 atm.

**Compound**	**k**_**OH**_	**k**_**Cl**_	**Class for Figure 3**
methane	6.40 × 10^−15^	1.00 × 10^−13^	Methane
ethane	2.40 × 10^−13^	5.90 × 10^−11^	Alkane
propane	1.10 × 10^−12^	1.40 × 10^−10^	Alkane
n-butane	2.36 × 10^−12^	2.18 × 10^−10^	Alkane
n-pentane	3.80 × 10^−12^	2.80 × 10^−10^	Alkane
i-butane	2.12 × 10^−12^	1.43 × 10^−10^	Alkane
i-pentane	3.60 × 10^−12^	2.20 × 10^−10^	Alkane
ethene	9.00 × 10^−12^	1.10 × 10^−10^	Alkene
propene	3.00 × 10^−11^	2.70 × 10^−10^	Alkene
1-butene	3.14 × 10^−11^	3.38 × 10^−10^	Alkene
trans-2-butene	6.40 × 10^−11^	3.31 × 10^−10^	Alkene
cis-2-butene	5.64 × 10^−11^	3.76 × 10^−10^	Alkene
CHCl_3_	1.00 × 10^−13^	1.20 × 10^−13^	Halocarbon
CHBr_3_	1.20 × 10^−13^	2.80 × 10^−13^	Halocarbon
CH_2_Cl_2_	1.00 × 10^−13^	3.50 × 10^−13^	Halocarbon
CH_2_Br_2_	1.80 × 10^−13^	3.80 × 10^−13^	Halocarbon
