# Supplementary material for: Evidence for strong, widespread chlorine radical chemistry associated with pollution outflow from continental Asia
Source: Sci Rep. 2016 Nov 15;6:36821. doi: 10.1038/srep36821 (PMC5109052; doi:10.1038/srep36821)
Supplement: Supplementary Information [file srep36821-s1.pdf]

Supplementary Information

Evidence for strong, widespread chlorine radical chemistry associated with pollution outflow from continental Asia

Authors: Angela K. Baker[1], Carina Sauvage[1], Ute R. Thorenz[1], Peter van Velthoven[2], David E. Oram[3], Andreas Zahn[4], Carl A. M. Brenninkmeijer[1] and Jonathan Williams[1*]

Affiliations:

[1]Max Planck Institute for Chemistry, Mainz, Germany

[2]Royal Netherlands Meteorological Institute (KNMI), De Bilt, the Netherlands

[3]National Centre for Atmospheric Science, University of East Anglia, Norwich, United Kingdom

[4]Karlsruhe Institute of Technology, Karlsruhe, Germany

*Correspondence to: Jonathan.Williams@mpic.de

Derivation of Equation 1 and basis for analysis calculations. Photochemical losses of an atmospheric species, A, attributable to a single oxidant, X, can be described by the equation

$$[A]_t = [A]_0 e^{-k_X \langle [X] \rangle \Delta t} \qquad \text{(S1)}$$

where $[A]_t$ is the concentration at time t, $[A]_0$ is the initial concentrations, k_X is the rate of reaction with oxidant X and [X] is the mean concentration of the oxidant during the transport time, Δt. Equation S1 can be rearranged to solve for any of the above parameters, and is often used as the basis for so-called "NMHC photochemical clock" methods (*11,41-44*), which are frequently employed to investigate transport times (Δt) or oxidant concentrations. Given the large variability in NMHC concentrations and corresponding uncertainty in their initial levels, these methods more commonly rely on NMHC ratios, comparing observed ratios with initial, typically emission, ratios, which tend to lie within narrow ranges and are relatively well-known. In order to reduce the influence of mixing, observed ratios, also referred to as enhancement ratios, are determined from the slopes of the linear least squares fit to the data. Changes in NMHC ratios with processing can be described by combining Equation S1 for two compounds, A and B (expressed here in terms of the natural logarithm):

$$ln\left(\frac{[A]_t}{[B]_t}\right) = ln\left(\frac{[A]_0}{[B]_0}\right) - (k_X^A - k_X^B)\langle [OH] \rangle \Delta \qquad \text{(S2)}$$

The further combination of Equation 2 for two different oxidants and NMHC pairs results in the time-independent expression used to derive oxidant ratios shown in Equation 1.

Possible influence of NO_3 on NMHC ratios. A $ClNO_2$ source of Cl radicals would also serve as a source of nitrate (NO_3) radicals, which also react with the NMHCs discussed here. NO_3 would also be expected to play a role in nighttime oxidation in regions having high levels of NO_x and O_3 (regardless of the presence of $ClNO_2$), as would be expected for polluted air masses

originating in continental Asia. However, reactions of alkanes with NO_3 proceed much more slowly than with Cl (or OH), on the order of 10^{-17}-10^{-16} cm^3 $molec^{-1}$ s^{-1} *(44)*, and would not be expected to be a dominant loss mechanism. More significantly, the reaction of NO_3 with i-butane is faster than with n-butane, so reaction with NO_3 would cause the ratio of i-butane/n-butane to decrease over time. A simple calculation where 10^9 NO_3 cm^{-3} is present as an oxidant in addition to the approximately 1.5×10^6 OH cm^{-3} and 2×10^4 Cl cm^{-3} estimated in this study shows that i-butane/n-butane would be about 4% lower after 7 days than if OH and Cl were the only oxidants. Ultimately, we find that NO_3 chemistry cannot explain the high ratios observed in this study, and would, in fact, serve to lower the ratios observed, therefore making our estimates of [Cl]:[OH] lower limits.

References

41. J. Rudolph, B. Ramacher, C. Plass-Dülmer, K. P. Müller, R. Koppmann, The indirect determination of chlorine atom concentration in the troposphere from changes in the patterns of non-methane hydrocarbons. *Tellus B* **49**, 592-601 (1997).
42. B. T. Jobson *et al.*, Spatial and temporal variability of nonmethane hydrocarbon mixing ratios and their relation to photochemical lifetime. *J. Geophys. Res.* **103**, 13557-13567 (1998).
43. D. D. Parrish *et al.*, Indications of photochemical histories of Pacific air masses from measurements of atmospheric trace species at Point Arena, California. *Journal of Geophysical Research: Atmospheres* **97**, 15883-15901 (1992).
44. R. Atkinson, Gas-phase tropospheric chemistry of volatile organic compounds: 1. Alkanes and alkenes. *J. Phys. Chem. Ref. Data* **26**, 215-290 (1997).
